# Notebook of experiences: a therapeutic resource in educational audiology

**DOI:** 10.1590/2317-1782/20212020422

**Published:** 2022-01-05

**Authors:** Natália Barreto Frederigue-Lopes, Débora Prevideli Soldera, Joice de Moura Silva, Camila Medina, Adriane Lima Mortari Moret, Regina Tangerino de Souza Jacob

**Affiliations:** 1 Departamento de Fonoaudiologia, Faculdade de Odontologia de Bauru – FOB, Universidade de São Paulo – USP - Bauru (SP), Brasil.

**Keywords:** Hearing Loss, Child, Hearing Aids, Rehabilitation, Cochlear Implantation

## Abstract

**Purpose:**

To translate the My Experience Book toll into Portuguese, evaluate the translation content, readability, quality, and visual identity of the material, and make it available online.

**Methods:**

Descriptive, cross-sectional, quantitative, and qualitative study. The procedures consisted of five stages: translation of the material; evaluation and response to questionnaire one regarding translation; determining the validity of the content; readability assessment; availability of material online and evaluation of content aimed at quality and visual identity (questionnaire two). Twenty-five professionals (audiologists and physicians) participated in the study.

**Results:**

The translation of the notebook showed validity agreement rates greater than 90%. Readability rated the material easy to read. Among the 184 invited professionals, only 25 agreed to participate, demonstrating low adherence to the study. Most respondents agreed positively about the content and consistency of the translated material, videos, illustrative images, and captions. The material was also considered necessary by the majority of the evaluators. All audiologists reported using the instrument in clinical practice.

**Conclusion:**

The translated toll is of great relevance. It gathers practical information to create a notebook of experiences and guidance on using the material as an additional resource to stimulate the auditory skills of children with hearing impairment.

## INTRODUCTION

Social interactions in children with typical hearing and language development are based on dialogue. In children with hearing loss, however, even with cognitive and affective maturity similar to their hearing peers, the dialogical hypotheses may be impaired due to the little contact and linguistic stimulation stimulated in the first years of life, resulting from auditory sensory deprivation and the presented speech unintelligibility. Scenarios like these result in difficulty in understanding the hypotheses reported by the child both in a therapeutic environment and in daily life^(1,2)^.

The family's participation in the therapeutic process enables knowledge of the children's routines, habits, preferences, facilities and difficulties^(3)^. Such information facilitates the understanding and attribution of meanings in the speech of children with a limited speech repertoire, being valuable to speech therapists in identifying the contexts in which the linguistic reports are inserted, as well as in the therapeutic planning with an offer of personalized intervention to the needs of the child and family with a view for expanding the dialogues and linguistic scenarios^(1-3)^.

Among the therapeutic strategies available for the development of auditory and oral language skills in a pleasurable, realistic and contextualized way for children, the use of specific tools, symbolic games and audiovisual resources, aimed at recording dialogues and daily social and family experiences stand out^(1,2)^.

In this context, the experience book emerged as an auxiliary material in the auditory rehabilitation process aimed at breaking down barriers and dialogical difficulties since it supports the child, while their language skills are not effective for maintaining dialogue^(1)^. When guided and built by family members, the experience book can help in different ways in child development for the acquisition and development of oral language, auditory function, as well as in the increase of communicative and dialogical bonds^(4,5)^.

Authors suggest that the creation of the experience notebook follow some criteria, namely, creativity, sequencing, playful, systematic and constructive^(6)^. As a result, it is essential to incorporate content related to children's daily life through the use of photos with family and friends, as well as pictures and collages that remind the child about the moments experienced in those contexts, transforming it into a diary of records of real and remarkable facts. This interaction strategy reveals the fundamental role of the family, not only in the preparation and construction of the material, but also in facilitating children's learning as a result of adherence and family involvement in the therapeutic process^(7)^.

When inserted in the context of the clinical speech therapy routine, the experience book provides the therapist with a possibility of efficient stimulation that is, at the same time, playful and fun, with the ability to help the patient in the construction of language, strengthening the dialogue between them^(8)^.

With the increase in chronological age, the development of auditory and oral language skills, and the strengthening of the relationships established between the patient, the therapist and the family, it is expected that children will mature in their ability to recognize, point out, name and speak on the elements, scenarios and situations based on the models provided by the mediating adults^(5)^.

Despite the value of the experience book strategy in the clinical speech therapy routine and in the development of children with hearing impairment, this subject is still little discussed in the specialized literature.

At the same time, the impact of technological advances on the number of people who use the internet in search of information and guides about their own health condition or that of family members is verified. This reality is even greater among family members who need ongoing therapeutic support for children with some type of disability, including hearing loss since such conditions evoke concerns and at the same time, arouse hope in the search for innovative treatments. In this sense, the internet becomes an important resource for the management of health care for children and adolescents, an alternative source of information and reducing anxiety for families involved in therapeutic processes^(9,10)^.

Aiming to provide scientific materials with content adequate to the needs of families of children with hearing impairment, speech therapy has been dedicated to building websites and providing online tools for the dissemination of instructional and guidance content, achieving motivating results for further research^(11)^. In this perspective, the responsibility to provide secure, reliable *online* information based on quality scientific sources and evidence becomes increasingly greater, considering the access of different audiences to the same information available^(12-14)^.

Therefore, the present study aimed to translate the *My Experience Book* tool into Portuguese, evaluate the content of the translation, the level of readability, quality and visual identity of the material and make it available *online*.

## METHODS

Descriptive, cross-sectional, quantitative and qualitative study approved by the Research Ethics Committee of the Bauru School of Dentistry, University of São Paulo (FOB/USP), under opinion CAAE nº 59790416.0.0000.5417.

**Figure 1:**
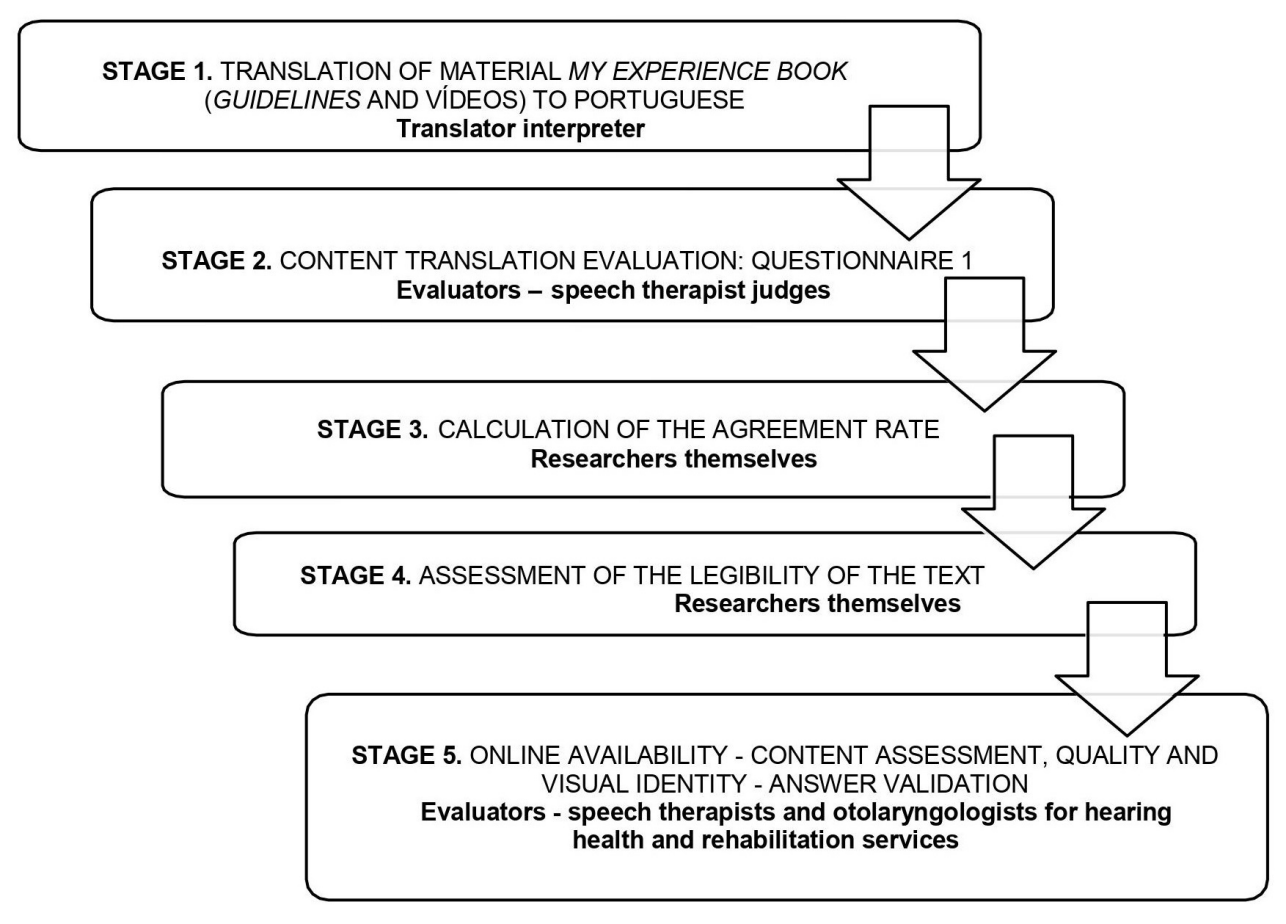
Description of the procedures for carrying out the present research

The procedures performed in the different stages of the research are described in Figure 1. The first stage consisted of translating the *My Experience Book*, a material consisting of texts, illustrations and videos on how and why to create an experience book developed by Nancy Caleffe-Schenck – Listen Foundation, available at the address: https://www.cochlear.com/intl/home/support/rehabilitation-resources/early-intervention/my-experience-book. The translation of the tool was authorized and carried out from English into Brazilian Portuguese by a translator-interpreter without prior knowledge of the material. The text was presented in PDF format and the videos were subtitled using the Windows Movie Maker program. The translated material was reviewed by the researchers in order to avoid technical terms, using words and expressions compatible with the target population.

Caption: Prepared by the authors

In the second stage, three speech-language pathologists-judges with experience in the area of Educational Audiology and Hearing Rehabilitation were invited to participate in the analysis of the translated material. Participants signed the Informed Consent Form (ICF) and then answered questionnaire 1: “Content evaluation: aspects related to translation,” developed by the researchers and made available on the *Google Forms* platform.

Next, to determine the validity of the content of the translation in a quantitative way, the agreement rate of the inter-observer responses was calculated based on the formula: % agreement = number of judges who agreed ÷ total number of judges X 100 (step 3). According to the calculation guidelines, the items become equivalent when the agreement rates exceed a percentage of 90%^(15)^.

The fourth stage included the evaluation of the readability of the translated text, using the Flesch Reading Ease Index (FREI) calculated using the *Microsoft Office Word* tool. According to the test, scores from 100 to 75 points are compatible with a very easy reading; 75 to 50 points - easy; 50 to 25 points – difficult; and 25 to 0 points - very difficult^(16)^.

Stage five consisted of making the material available to the evaluators in an *online* environment. Next, 184 professionals who graduated from the distance Specialization Course in Hearing Qualification and Rehabilitation in Children (SCHQRC), promoted by FOB-USP in partnership with the Ministry of Health and the Samaritano Hospital of São Paulo were invited to evaluate the content regarding the quality and visual identity with 175 (95.2%) speech therapists and nine (4.8%) otorhinolaryngologists working in hearing health and rehabilitation services. The invitation was made via e-mail containing the consent form, the links to access the *online* material and guidelines for completing the questionnaire 2: “Content evaluation: aspects related to quality and visual identity”, developed by the researchers, and available in the *Survey Monkey* tool. Invitations were resent three times to each professional and a period of 30 days was made available to complete participation in the research.

The validation of the invited professionals' responses regarding the content, quality and relevance of the translated material was performed using the Content Validity Index (CVI)^(17)^. According to the criteria used by the formula available in the literature, the calculation of the CVI must occur from the sum of the percentage of items marked by the participants as “4” or “5”, divided by the total number of questions. To obtain a valid CVI classification, a minimum percentage of agreement of 90% is required^(18)^.

Data analyses were organized in the *online* data collection tool itself. Descriptive statistics and analysis of the frequency of responses were used.

## RESULTS

**Figure 2:**
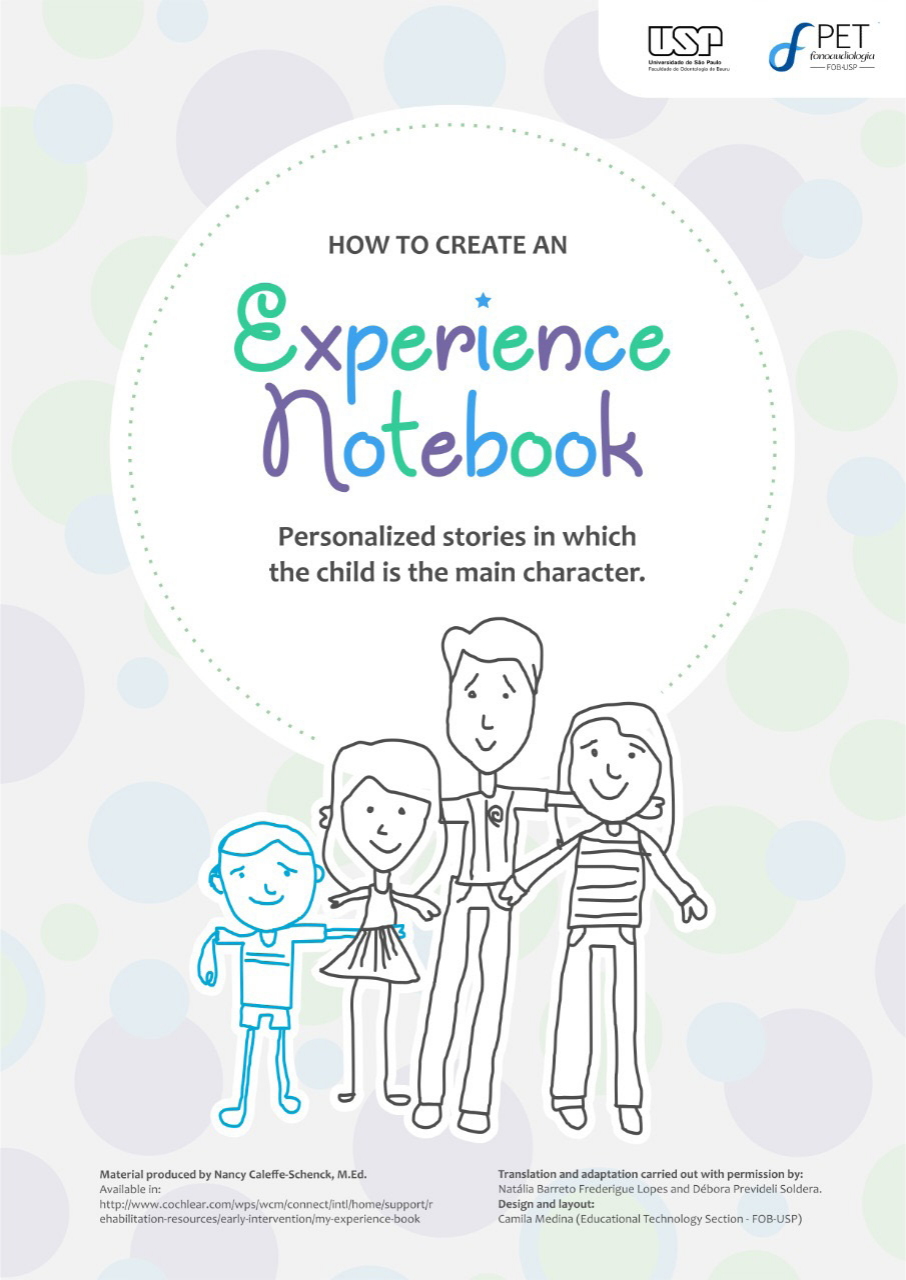
Example cover of tool

**Figure 3:**
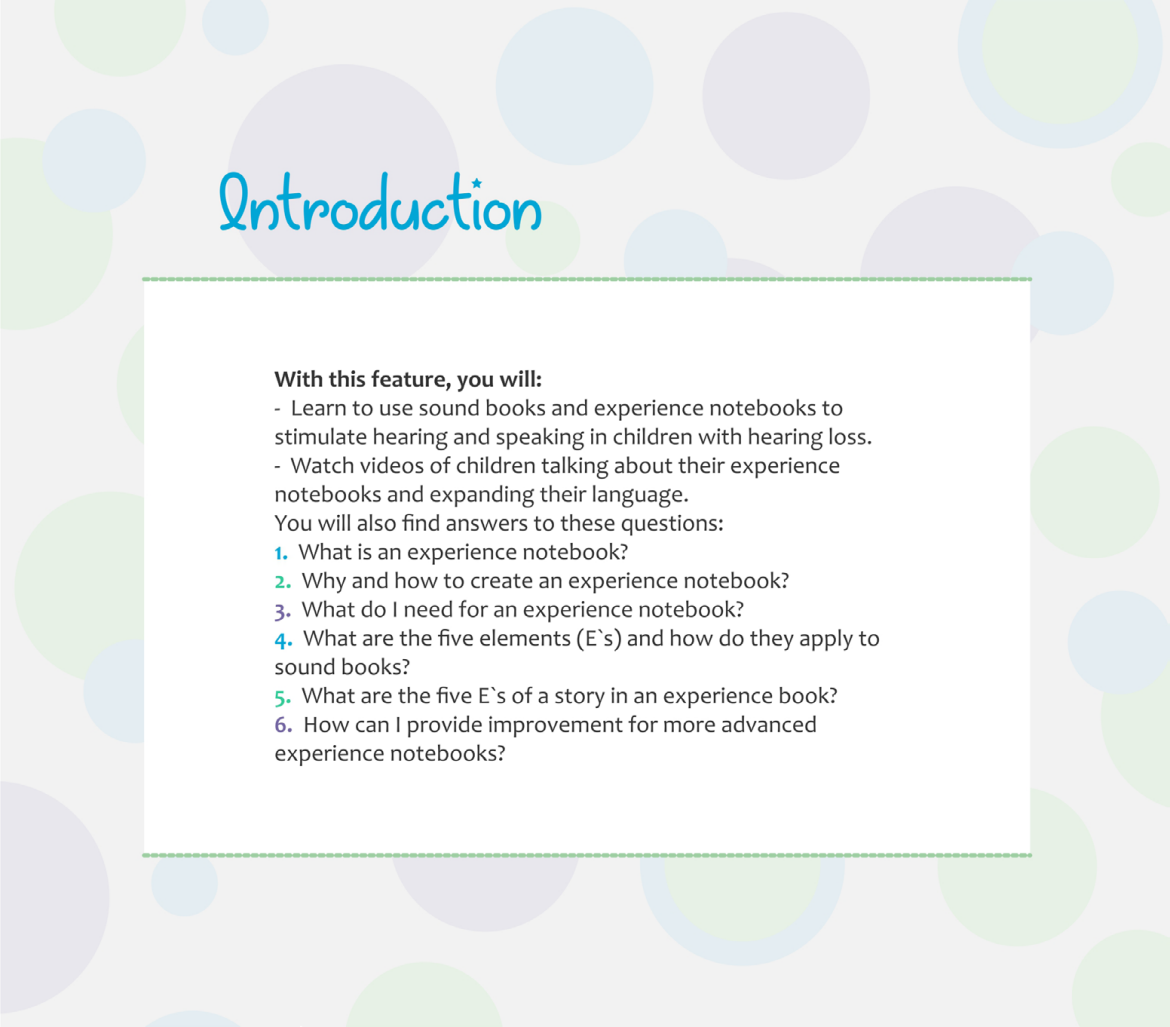
Tool summary example

The translated material was entitled “How to create a Notebook of Experiences: personalized stories in which the child is the main character” and organized in PDF format with a total of six pages (Figures 2 and 3). The graphic and textual content was organized according to the visual identity appropriate to the target audience (such as the choice of colors, illustrations and typography) in order to make the educational graphic material in question more engaging and providing greater engagement and motivation for the treatment. The videos received the titles: 1 – “The five E's and a notebook of experiences” (5:22 min); 2 – “Elements of a story in an experience notebook” (4:25 min); 3 – “Enrichment for an experience notebook” (5:57 min) and 4 – “Amartya's Pizza” (7:02 min).

Caption: Prepared by the authors

Caption: Prepared by the authors

The results obtained from the application of questionnaire 1, as well as the agreement rate of the validity of the content analysis are shown in Table 1.

**Table 1:** Answers regarding the evaluation of the translation of the content and validity agreement rate (n=3)

**Question**	**Answers**	**n**	**%**	**% Agreement**
Did you already know the material *My Experience Book*?	Yes	1	33.3	Not measurable
	No	2	66.7	
When comparing the content in English and in Portuguese, do you consider that the translation was: unsatisfactory, partially satisfactory or satisfactory?	Satisfatory	3	100.0	100.0
The translation of the PDF (Guidelines) presented itself as inviting for people to be interested in the audiovisual content?	Yes	3	100.0	100.0
Is the translation in an adequate language that is easy to understand for the population?	Yes	2	66.7	66.7
	No	1	33.3	
Were the audiovisual resources (videos) satisfactory in terms of quality? (final content will be made available with subtitles)	Yes	3	100.0	100.0
Is the translation of the videos self-explanatory and easy to understand on how to create an “experience notebook?”	Yes	3	100.0	100.0
Is the translated material inviting to the target audience?	Yes	3	100.0	100.0
Is the translation of the material as a whole effective in creating an “experience notebook” that helps in stimulating the children's language during the therapeutic process?	Yes	3	100.0	100.0

Caption: n = number; % = percentage

Source: Prepared by the authors

The initial level of readability of the text obtained by the FREI statistics was 42 points, classifying the material as “difficult.”^(16)^ After replacing the technical terms and reducing the number of words in the longer sentences, a score of 55 was obtained on the FREI, classifying the text as “easy.”

Even after sending invitations to participate in the survey and answering questionnaire 2, of the 184 guests, a total of 162 (88.04%) professionals did not access the *link* available on the *Survey Monkey* platform, while 22 (11.96%) did . Of these, four (2.18%) did not agree to participate in the research and only 18 (9.78%) expressed acceptance, being: 16 (8.69%) speech therapists and two (1.09%) otorhinolaryngologists.

The average time to complete the questionnaire was 21 minutes. The characterization of the profile of the participants is presented in Table 2.

**Table 2:** Characterization of research participants (n=18)

**Information**	**Description**	**n**	**%**
Age	31 to 55	M = 39.1 years	-
Sex	Feminine	16	88.8
	Masculine	2	11,2
Region of professional activity	North	5	27.8
	Northeast	4	22.2
	South	3	16.6
	Southeast	5	27.8
	Midwest	1	5.6
Work with speech therapy for children and/or adults with hearing impairment	Did not work	5	27.8
	Worked with children	5	27.8
	Worked with children and adults	8	44.4
Places of performance	Hospitals	2	11.1
	Clinics	10	55.5
	Universities	3	16.7
	Others	3	16.7
Knowledge about the content evaluated	Yes	8	44.4
	No	2	11.2
	Partially	8	44.4

Caption: n = number; % = percentage; M = mean

Source: Prepared by the authors

Table 3 shows the results regarding the evaluation of the content of the experience book.

**Table 3:** Answers regarding the evaluation of the content of the translated material (n=18)

**Questions**	**Totally disagree**		**Disagree**		**Indifferent**		**Agree**		**Totally agree**	
	**n**	**%**	**n**	**%**	**n**	**%**	**n**	**%**	**n**	**%**
Considering that the target audiences are speech therapists and family members of children with HI, do you consider that the text presented in the PDF is intelligible/understandable?	0	0.0	0	0.0	1	5.5	10	55.6	7	38.9
Is the content logically arranged, making it easier to understand?	0	0.0	1	5.5	0	0.0	12	66.7	5	27.8
Is the information offered enough for the elaboration of an experience notebook in the routine of children with hearing impairment?	0	0.0	0	0.0	2	11.1	9	50.0	7	38.9
Are the set of images, figures and videos relevant to the content?	0	0,0	0	0,0	0	0,0	10	55,6	8	44,4

Caption: n = number; % = percentage

Source: Prepared by the authors

Table 4 shows the assessments regarding the analysis of the visual quality of the translated material - PDF, videos, illustrative images and subtitles.

**Table 4:** Answers regarding the quality assessment of the translated material - PDF, videos, illustrative images and subtitles (n=18)

**Questions**	**Terrible**		**Bad**		**Regular**		**Good**		**Excellent**	
	**n**	**%**	**n**	**%**	**n**	**%**	**n**	**%**	**n**	**%**
How would you rate the quality of this material (PDF and videos)?	0	0.0	0	0.0	0	0.0	7	38.9	11	61.1
How would you rate the quality of the illustrative images of the content made available in PDF?	0	0.0	0	0.0	1	5.5	9	50.0	8	44.5
How would you rate the quality of the videos and their respective subtitles?	0	0.0	0	0.0	2	11.1	11	61.2	5	27.7

Caption: n = number; % = percentage

Source: Prepared by the authors

The importance of the experience notebook in clinical routine is described in Table 5.

**Table 5:** Answers regarding the importance of the material (n=18)

**Question**	**Not important at all**		**Not very important**		**Reasonably important**		**Very important**		**Extremely important**	
	**n**	**%**	**n**	**%**	**n**	**%**	**n**	**%**	**n**	**%**
How would you classify the elaborated material?	0	0.0	0	0.0	1	5.5	7	38.9	10	55.6

Caption: n = number; % = percentage

Source: Prepared by the authors

In the present study, the percentage of agreement in the Content Validity Index (CVI) was 94.48%.

## DISCUSSION

The experience book presented in the present article aimed to offer professionals in the field of speech therapy one more possibility of therapeutic resource aimed at the development of hearing and language skills of children with hearing impairment in an accessible, free and *online* way. We also sought to encourage knowledge, access, interest, adherence and family involvement in the therapeutic process both in the clinical and home environments based on the bonds built between therapist, patient and family, resulting from the strategies explored by through the experience notebook.

The evaluation of the validity of materials in the health area aims to facilitate the work of professionals with regard to health promotion, the guidance and support provided to patients and families, with a view to offering more effective conditions of care, treatment and rehabilitation of the target population^(19)^. The translation of the material was well evaluated by the judges with validity agreement rates greater than 90%, as indicated by the test used^(17)^. These rates apply both to the aspects of language analysis, as well as the ease of understanding, the attractiveness of the instrument, the quality of audiovisual resources and the tool as a whole (Table 1).

The specialized literature points out some relevant factors to carry out the validation of materials in the health area, among them, the possibility of obtaining more complete, effective instruments and with greater scientific rigor, since the opinion of judges contributes to the improvement of quality of the material^(20)^. In the present study, the additional suggestions acquired through questionnaire 1, considered relevant, were accepted and adjusted in the translated tool.

The information available in health instructional materials only becomes effective if understood by the target audience^(21)^. For this, it is necessary that the text presents a level of readability compatible with the cognitive abilities of the reader. Thus, when producing materials for such purposes, it is of paramount importance that health professionals direct special attention to the use of resources that facilitate reading and understanding the text, maximizing the reader's interest, such as the selection and use of words, technical expressions and meanings^(22)^. The quality of texts available in the *online* environment demand even greater care considering the possibility and speed of reaching a variety of audiences with different ages, education and socioeconomic levels^(13)^. Additionally, in these cases, it is the responsibility of speech therapists to publish safe, reliable information based on quality scientific sources and evidence^(12-14)^. Analysis of the readability of the translated text in the experience notebook classified the material as an easy-to-read level.

The use of *online* questionnaires as a data collection instrument has become a current trend, increasingly present in the scientific community, standing out as a preference both among researchers and participants^(23)^. Among the advantages of using this material are: global reach, flexibility, time savings, technological innovations, ease of data collection and tabulation, low cost, obtaining large samples and filling control mandatory. On the other hand, disadvantages are highlighted: the presence of spam, the selection and quality of the sample, the lack of skills of respondents, the need for technological resources, impersonality and low adherence^(24)^. Regarding the last factor, the literature indicates that questionnaires in *online* format reach, on average, 25% of adherence^(25)^.

Low adherence was seen in the present research. Despite the ease of access and the experience of the guests regarding the use of the internet, the return rate of professionals showed a limitation to the results, even after re-sending the invitations (three times in total), adherence to the answer to questionnaire 2, was only 22 (11.96%) participants. The justification for low adherence may be based on the lack of interest and participation of the guests, in addition to the time needed to evaluate the content, which included reading the material in PDF (six pages), analyzing the videos (22 .06 minutes), and the answer to the questionnaire (average of 21 minutes). Despite the low participation of invited professionals, the Content Validity Index obtained revealed a percentage of 94.48%, indicating a reliable relationship between the number of respondents and the responses obtained.

Similar results have been identified in other internet-related searches. A study^(26)^ investigated the competences of serving nurses in educational institutions regarding the performance of essential public health functions, and pointed out that, of the 31 invited universities, only six (19.35%) returned contact with an indication of professional nursing experts. Of these, only one nurse (16.66%) responded to the proposed form. Another research proposed the application of an *online* questionnaire with technical-administrative employees and heads of education. In this one, the response rates obtained were 20.78% (272) for the 1,309 employees invited, and 13.85% (9) for the bosses who responded to the questionnaire^(27)^.

Regarding the content and coherence of the translated material, videos and thematic addressed, intelligibility and layout of the text, audiovisual resources and information for the composition of the experience book, most evaluators responded that they agreed or totally agreed with the investigated aspects (Table 3). In an additional dissertation analysis, the participants judged the material as practical, accessible and easily understood by the public due to the approach of the different phases and possibilities for an effective stimulation. In general, the aforementioned aspects can be summarized in three main points: accessibility, ease of understanding and use of the material. Regarding this, the literature points out that health guidance content made available *online* can serve as an additional resource for stimulating the child, favoring therapeutic support networks and adding or disseminating new knowledge. Such aspects are valuable to guarantee the active participation of the family in the therapeutic process^(28)^.

In the present research, most participants considered the quality of the translated material - PDF, videos, illustrative images and subtitles as good or excellent (Table 4). Furthermore, in a dissertation manner, they pointed out that the good quality, illustration and visibility of the images facilitated understanding and made reading more pleasurable. The videos, in turn, were described as well produced, with good quality and presentation of practical and real situations, which enriched the entire material. Knowing that the literature recognizes that the use of illustrations, videos and images can favor the understanding, internalization and learning of new content^(29)^, it is considered that the way the material is organized can influence adherence to use by the target audience.

Regarding the importance of the material, 94.5% (n=17) considered the experience book to be very or extremely important (Table 5). In addition, all speech therapist participants reported using the instrument in clinical practice. The therapists added that this therapeutic resource is very important and useful since it provides the structuring of oral and written language stimulates creativity and provides an increase in family relationships with the possibility of use in all phases of therapy. Authors indicate that additional tools aimed at promoting the communicative development of children with hearing impairment, which enable them to adapt to the specific needs of each individual, such as the experience book, act as materials rich in stimulation, as they adjust to different contexts experienced by the child^(30)^. It is noteworthy that the potential of using the experience book in speech therapy is not restricted to children with hearing impairment, but can be used in the therapeutic process of children with other alterations, for example, language disorders^(2)^.

## CONCLUSION

The tool “How to create an experience notebook: personalized stories in which the child is the main character”, was evaluated as adequate in terms of translation with a high rate of validity agreement. Readability revealed an easy readability level. Most evaluators rated the quality and visual identity of the content as excellent, extremely important and suitable for *online* availability.

Such material is of great relevance since it gathers practical information aimed at creating an experience notebook, as well as guidance on the use of the material as an additional resource for stimulating the development of auditory and language skills in children with hearing impairment.

It is expected that from the present study, speech therapists, health and education professionals, parents and/or guardians and other interested parties will have access to this valuable therapeutic tool *online* and free of charge through the Hearing now and Always website from Cochlear®.

## References

[1] Melo EM (2000). Caderno de experiências no processo terapêutico da criança portadora de deficiência auditiva.

[2] Melo ME, Novaes BCAC (2001). Caderno de experiências no processo terapêutico de uma criança portadora de deficiência auditiva. Pro Fono.

[3] Abbud GAC, Santos TCES (2002). A família na clínica fonoaudiológica e psicopedagógica: uma valiosa parceria. Psicol Teor Prat.

[4] Schwartz S, Miller JEH (1996). The new language of toys: teaching communication skills to children with special needs: a guide for parents and teachers..

[5] Pollack D, Goldberg D, Caleffe-schenck N (1997). Educational audiology for the limited-hearing infant and preschooler: an Auditory-Verbal Program..

[6] Rigolet SAN, Costa IMM (1995). Estimulação precoce da linguagem escrita e o uso do caderno diário.

[7] Beebe HH, Person HR, Koch ME, Ling D (1984). Early intervention for hearing-impaired children: oral options. Houlton.

[8] Estabrooks W (1994). Auditory-verbal therapy for parents and professionals..

[9] Hand F, McDowell DT, Glynn RW, Rowley H, Mortell AF (2013). Patterns of internet use by parents of children attending a pediatric surgical service. Pediatr Surg Int.

[10] Lee K, Hoti K, Hughes JD, Emmerton L (2014). Dr Google and the consumer: a qualitative study exploring the navigational needs and online health informationseeking behaviors of consumers with chronic health conditions. J Med Internet Res.

[11] Blasca WQ, Maximino LP, Galdino DG, Campos K, Picolini MM (2010). Novas tecnologias educacionais no ensino da audiologia. Rev CEFAC.

[12] Corrêa CC, Silva RA, Blasca WQ (2014). Elaboration and evaluation of contents about hearing health inserted in cybertutor. Int Arch Otorhinolaryngol.

[13] Chaves JN, Libardi AL, Agostinho-Pesse RS, Morettin M, Alvarenga KF (2015). Telessaúde: avaliação de websites sobre triagem auditiva neonatal na Língua Portuguesa. CoDAS.

[14] Maximino LP, Zambonato TCF, Picolini-Pereira MM, Corrêa CC, Feniman MR, Blasca WQ (2018). Development and evaluation of a blog about cleft lip and cleft palate and hearing. Int Arch Otorhinolaryngol.

[15] Wynd CA, Schmidt B, Schaefer MA (2003). Two quantitative approaches for estimating content validity. West J Nurs Res.

[16] Silva CAT, Fernandes JLT (2009). Legibilidade dos fatos relevantes no Brasil. RAC-Eletrônica..

[17] Polit DF, Beck CT (2006). The content validity index: are you sure you know what’s being reported? Critique and recomendations. Res Nurs Health.

[18] Alexandre MC, Coluci MZ (2011). Validade de conteúdo nos processos de construção e adaptação de instrumentos de medidas. Cien Saude Colet..

[19] Áfio ACE, Balbino AC, Alves MDS, Carvalho LV, Santos MCL, Oliveira NR (2014). Analysis of the concept of nursing educational technology applied to the patient. Rev Rene..

[20] Polit DF, Beck CT (2006). The Content Validity Index: are you sure you know what’s being reported? Critique and recommendations. Res Nurs Health.

[21] Hoffmann T, Worrall L (2004). Designing effective written health education materials: considerations for health professionals. Disabil Rehabil.

[22] Friedman DB, Hoffman-Goetz L (2006). A systematic review of readability and comprehension instruments used for print and web-based cancer information. Health Educ Behav.

[23] Zuidgeest M, Hendriks M, Koopman L, Spreeuwenberg P, Rademakers J (2011). A comparison of a postal survey and mixed-mode survey using a questionnaire on patients’ experiences with breast care. J Med Internet Res.

[24] Gonçalves DIF (2008). Pesquisas de marketing pela internet: as percepções sob ótica dos entrevistados. Rev Adm Mackenzie..

[25] Marconi MA, Lakatos EM (2005). Fundamentos de metodologia científica..

[26] Silva AM, Rodrigues CDS, Silva SMR, Witt RR (2009). Utilização da técnica Delphi on-line para investigação de competências: relato de experiência. Rev Gaúcha Enferm.

[27] Reorganiza UFSC (2013). Isonomia para todos: relatório final. Grupo de Trabalho Reorganiza UFSC: isonomia para todos..

[28] Shoham S, Heber M (2012). Characteristics of a virtual community for individuals who are d/deaf and hard of hearing. Am Ann Deaf.

[29] Hayden P (2005). Learner’s pocketbooks..

[30] Veenema S, Gardner H (1996). Multimedia and multiple inteligences. Am Prospect.

