# Supplementary material for: Notebook of experiences: a therapeutic resource in educational audiology
Source: Codas. 2022 Jan 5;34(1):e20200422. doi: 10.1590/2317-1782/20212020422 (PMC9886119; doi:10.1590/2317-1782/20212020422)
Supplement: Translation

# Translation: Caderno de experiências: um recurso terapêutico na audiologia educacional

**Authors:** Natália Barreto Frederigue-Lopes, Débora Prevideli Soldera, Joice de Moura Silva, Camila Medina, Adriane Lima Mortari Moret, Regina Tangerino de Souza Jacob

**Affiliations:** 1 Departamento de Fonoaudiologia, Faculdade de Odontologia de Bauru – FOB, Universidade de São Paulo – USP - Bauru (SP), Brasil.

**Keywords:** Perda Auditiva, Criança, Auxiliares de Audição, Reabilitação, Implante Coclear

## Abstract

**Objetivo:**

Traduzir para o idioma português a ferramenta *My Experience Book*, avaliar o conteúdo da tradução, o nível de legibilidade, a qualidade e a identidade visual do material e disponibilizá-lo *online*.

**Método:**

Estudo descritivo, transversal, de caráter quantitativo e qualitativo. Os procedimentos contaram com cinco etapas, sendo: tradução do material; avaliação e resposta ao questionário 1 referente à tradução; determinação da validade do conteúdo; avaliação da legibilidade; disponibilização do material online e avaliação do conteúdo voltado à qualidade e a identidade visual (questionário 2). Participaram do estudo três fonoaudiólogos, juízes e 22 profissionais (fonoaudiólogos e médicos otorrinolaringologistas de serviços de saúde auditiva e reabilitação).

**Resultados:**

A tradução do material apresentou taxas de concordância de validade maiores que 90%. A legibilidade classificou o material com nível fácil de leitura. Dentre os 184 profissionais convidados, 22 concordaram com a participação, demonstrando baixa adesão ao estudo. A maior parte dos respondentes concordaram positivamente com os aspectos relacionados ao conteúdo e coerência do material traduzido, vídeos, imagens ilustrativas e legendas. O material também foi considerado importante pela maioria dos avaliadores. Todos os fonoaudiólogos referiram fazer uso do instrumento na prática clínica.

**Conclusão:**

O material traduzido referente à ferramenta é de grande relevância, pois reúne informações práticas voltadas à criação de um caderno de experiências, bem como orientações quanto ao uso do instrumento como recurso adicional para a estimulação do desenvolvimento das habilidades auditivas e de linguagem de crianças com deficiência auditiva.

## INTRODUÇÃO

As interações sociais em crianças com desenvolvimento típico de audição e de linguagem baseiam-se em diálogos. Na criança com deficiência auditiva, entretanto, mesmo com a maturidade cognitiva e afetiva semelhantes aos seus pares ouvintes, as situações dialógicas podem apresentar-se prejudicadas, devido ao pouco contato e estímulo linguístico recebido nos primeiros anos de vida, decorrentes da privação sensorial auditiva e da ininteligibilidade de fala apresentada. Cenários como estes, resultam em dificuldade na compreensão das situações relatadas pela criança tanto em ambiente terapêutico quanto na vida diária^(1,2)^.

A participação da família no processo terapêutico possibilita o conhecimento das rotinas, hábitos, preferências, facilidades e dificuldades das crianças^3^. Tais informações facilitam a compreensão e a atribuição de significados em discurso de crianças com escasso repertório de fala, sendo valiosas aos fonoaudiólogos na identificação dos contextos aos quais os relatos linguísticos encontram-se inseridos, bem como ao planejamento terapêutico com oferta de intervenção personalizada às necessidades individuais da criança e da família, tendo em vista à ampliação dos diálogos e dos cenários linguísticos^(1-3)^.

Dentre as estratégias terapêuticas disponíveis para o desenvolvimento das habilidades auditivas e de linguagem oral de forma prazerosa, realista e contextualizada, destacam-se o uso de ferramentas específicas, jogos simbólicos e recursos audiovisuais, voltados ao registro de diálogos e as experiências sociais e familiares diárias das crianças^(1,2)^.

Neste contexto, o caderno de experiências surgiu como um material auxiliar no processo de reabilitação auditiva voltado ao rompimento das barreiras e das dificuldades dialógicas, uma vez que serve de apoio para a criança, enquanto suas habilidades linguísticas não são eficazes para a manutenção do diálogo^(1)^. Quando orientado e construído pelos familiares, o caderno de experiências pode auxiliar de diferentes maneiras no desenvolvimento infantil, na aquisição e desenvolvimento da linguagem oral, da função auditiva, bem como no aumento dos laços comunicativos e dialógicos^(4,5)^.

Autores sugerem que a criação do caderno de experiências siga alguns critérios, sendo eles, a criatividade, a sequencialização, o lúdico, o sistemático e o construtivo^(6)^. Para isso, é fundamental a incorporação de conteúdos relacionados ao cotidiano infantil por meio do uso de fotos com a família e amigos, além de figuras e colagens que relembrem a criança sobre os momentos vivenciados naqueles contextos, transformando-o em um diário de registros de fatos reais e marcantes. Esta estratégia de interação revela o papel fundamental da família, não só na elaboração e construção do material mas, também, na facilitação da aprendizagem das crianças, como resultado da adesão e do envolvimento familiar no processo terapêutico^(7)^.

Quando inserido no contexto de rotina clínica fonoaudiológica, o caderno de experiências fornece ao terapeuta uma possibilidade de estimulação eficiente e, ao mesmo tempo, lúdica e divertida, com capacidade de auxiliar o paciente na construção da linguagem, fortalecendo o diálogo entre ambos^(8)^.

Com o aumento da idade cronológica, o desenvolvimento das habilidades auditivas, de linguagem oral, e o fortalecimento das relações estabelecidas entre o paciente, o terapeuta e a família, espera-se que as crianças amadureçam as capacidades de reconhecer, apontar, nomear e discursar sobre os elementos, cenários e situações, com base nos modelos fornecidos pelos adultos mediadores^(5)^.

Apesar do valor da estratégia do caderno de experiências na rotina clínica fonoaudiológica e no desenvolvimento de crianças com deficiência auditiva, este assunto ainda é pouco discutido pela literatura especializada.

Paralelamente, verifica-se o impacto do avanço tecnológico sobre a quantidade de pessoas que utilizam a internet na busca por informações e guias sobre a própria condição de saúde ou de familiares. Tal realidade é ainda maior entre familiares que necessitam de apoio terapêutico contínuo para crianças com algum tipo de deficiência, incluindo a deficiência auditiva, visto que, tais condições evocam inquietudes e, ao mesmo tempo, despertam esperanças na busca por tratamentos inovadores. Neste sentido, a internet torna-se um importante recurso para o gerenciamento do cuidado à saúde infanto-juvenil, fonte alternativa de informações e redução da ansiedade das famílias inseridas em processos terapêuticos^(9,10)^.

Visando fornecer materiais científicos com conteúdo adequado às necessidades das famílias de crianças com deficiência auditiva, a fonoaudiologia tem se dedicado à construção de *websites* e a disponibilização de ferramentas *online* para a disseminação de conteúdos instrucionais e de orientação, alcançando resultados motivadores para novas pesquisas^(11)^. Nesta perspectiva, a responsabilidade de prover informações *online* seguras, confiáveis e fundamentadas em fontes e evidências científicas de qualidade, torna-se cada vez maior, considerando o acesso de diferentes públicos à mesma informação disponibilizada^(12-14)^.

Diante disso, este estudo objetivou traduzir para o idioma português a ferramenta *My Experience Book*, avaliar o conteúdo da tradução, o nível de legibilidade, a qualidade e a identidade visual do material e disponibilizá-lo *online*.

## MÉTODO

Estudo descritivo, transversal, de caráter quantitativo e qualitativo, aprovado pelo Comitê de Ética em Pesquisa da Faculdade de Odontologia de Bauru da Universidade de São Paulo (FOB/USP), sob o parecer CAAE nº 59790416.0.0000.5417.

Os procedimentos realizados nas diferentes etapas da pesquisa estão descritos na Figura 1. A primeira etapa foi composta pela tradução do *My Experience Book*, um material formado por textos, ilustrações e vídeos de como, e por que, criar um caderno de experiências, desenvolvido por Nancy Caleffe-Schenck – *Listen Foundation*, disponível no endereço: https://www.cochlear.com/intl/home/support/rehabilitation-resources/early-intervention/my-experience-book. A tradução da ferramenta foi autorizada e realizada do inglês para o português brasileiro, por um tradutor-intérprete sem conhecimento prévio do material. O texto foi apresentado em formato PDF e os vídeos legendados por meio do programa *Windows Movie Maker*. O material traduzido foi revisado pelos pesquisadores de forma a evitar termos técnicos, utilizar vocábulos e expressões compatíveis à população alvo.

**Figura 1:** Descrição dos procedimentos para a realização desta pesquisa

Fonte: Elaborado pelos autores

Na segunda etapa, três fonoaudiólogos-juízes com experiência na área da Audiologia Educacional e Reabilitação Auditiva foram convidados a participar da análise do material traduzido. Os participantes assinaram o Termo de Consentimento Livre e Esclarecido (TCLE) e, em seguida, responderam ao questionário 1: “Avaliação do conteúdo: aspectos referentes à tradução”, desenvolvido pelos pesquisadores e disponibilizado na plataforma *Google Forms*.

Em seguida, para determinar a validade do conteúdo da tradução em caráter quantitativo, calculou-se a taxa de concordância das respostas inter-observadores com base na fórmula: % de concordância = número de juízes que concordaram ÷ número de total de juízes X 100 (etapa 3). Conforme as orientações do cálculo, os itens tornam-se equivalentes, quando as taxas de concordância ultrapassam um percentual de 90%^(15)^.

A quarta etapa contemplou a avaliação da legibilidade do texto traduzido, por meio do Índice de Facilidade de Leitura Flesch (IFLF) calculado na ferramenta do *Microsoft Office Word*. Segundo o teste, pontuações de 100 à 75 pontos são compatíveis com uma leitura muito fácil; 75 à 50 pontos - fácil; 50 à 25 pontos – difícil; e 25 à 0 pontos - muito difícil^(16)^.

A etapa cinco consistiu na disponibilização do material aos avaliadores em ambiente *online*. Em seguida, 184 profissionais egressos do Curso de Especialização a distância em Habilitação e Reabilitação Auditiva em Crianças (CEDHRAC), promovido pela FOB-USP em parceria com o Ministério da Saúde e Hospital Samaritano de São Paulo, foram convidados a avaliar o conteúdo quanto à qualidade e identidade visual, sendo 175 (95,2%) fonoaudiólogos e nove (4,8%) médicos otorrinolaringologistas, atuantes em serviços de saúde auditiva e de reabilitação. O convite foi realizado via e-mail contendo o TCLE, os *links* de acesso ao material *online* e as orientações de preenchimento do questionário 2: “Avaliação do conteúdo: aspectos referentes à qualidade e a identidade visual”, desenvolvido pelos pesquisadores, e disponibilizado na ferramenta *Survey Monkey*. Os convites foram reenviados três vezes a cada profissional e disponibilizado um período de 30 dias para a conclusão da participação na pesquisa.

A validação das respostas dos profissionais convidados, referentes ao conteúdo, qualidade e relevância do material traduzido, foi realizada por meio do Índice de Validade de Conteúdo (IVC)^(17)^. De acordo com os critérios utilizados pela fórmula disponibilizada na literatura, o cálculo do IVC deve ocorrer a partir da soma da porcentagem dos itens marcados pelos participantes como “4” ou “5”, dividido pelo número total de questões. Para a obtenção de uma classificação válida de IVC, é necessário um percentual mínimo de concordância de 90%^(18)^.

As análises dos dados foram organizadas na própria ferramenta *online* de coleta dos dados. Utilizou-se estatística descritiva e análise da frequência das respostas.

## RESULTADOS

O material traduzido foi intitulado “Como criar um Caderno de Experiências: histórias personalizadas em que a criança é a personagem principal” e organizado em formato PDF, com o total de seis páginas (Figuras 2 e 3). O conteúdo gráfico e textual foi organizado conforme identidade visual apropriada ao público alvo (como a escolha das cores, ilustrações e tipografia) de forma a tornar o material gráfico educacional em questão mais envolvente e que proporcione maior engajamento e motivação ao tratamento. Os vídeos receberam os títulos: 1 – “Os cinco E’s e um caderno de experiências” (5:22 min); 2 – “Elementos de uma história em um caderno de experiências” (4:25 min); 3 – “Enriquecimento para um caderno de experiência” (5:57 min) e 4 – “A Pizza de Amartya” (7:02 min).

**Figura 2:** Exemplo da capa da ferramenta

Fonte: Elaborado pelos autores

**Figura 3:** Exemplo do sumário da ferramenta

Fonte: Elaborado pelos autores

Os resultados obtidos a partir da aplicação do questionário 1, bem como a taxa de concordância da validade da análise do conteúdo estão dispostos na Tabela 1.

**Tabela 1:** Respostas referentes à avaliação da tradução do conteúdo e taxa de concordância da validade (n=3)

**Questão**	**Respostas**	**n**	**%**	**% Concordância**
Você já conhecia o material *My Experience Book*?	Sim	1	33,3	Não mensurável
	Não	2	66,7	
Ao comparar o conteúdo em inglês e em português, você considera que a tradução se apresentou: insatisfatória, parcialmente satisfatória ou satisfatória?	Satisfatória	3	100,0	100,0
A tradução do PDF (*Guidelines*) se apresentou convidativa para que as pessoas se interessem pelos conteúdos audiovisuais?	Sim	3	100,0	100,0
A tradução se encontra com linguagem adequada e de fácil entendimento para a população?	Sim	2	66,7	66,7
	Não	1	33,3	
Os recursos audiovisuais (vídeos) foram satisfatórios quanto à qualidade? (o conteúdo final será disponibilizado com legendas)	Sim	3	100,0	100,0
A tradução dos vídeos é autoexplicativa e de fácil entendimento de como se criar um “caderno de experiências”?	Sim	3	100,0	100,0
O material traduzido é convidativo para o público alvo?	Sim	3	100,0	100,0
A tradução do material como um todo é efetiva para a criação de um “caderno de experiências” que auxilie na estimulação de linguagem das crianças durante o processo terapêutico?	Sim	3	100,0	100,0

Legenda: n = número; % = porcentagem

Fonte: Elaborado pelos autores

O nível inicial de legibilidade do texto obtido pela estatística IFLF foi de 42 pontos, classificando o material como “difícil”^(16)^. Após a substituição dos termos técnicos e a redução do número de palavras das sentenças mais longas, obteve-se pontuação de 55 no IFLF, classificando o texto como “fácil”.

Mesmo após o reenvio dos convites para participação na pesquisa e resposta ao questionário 2, dos 184 convidados, um total de 162 (88,04%) profissionais não acessaram o *link* disponibilizado na plataforma *Survey Monkey*, enquanto 22 (11,96%) acessaram. Destes, quatro (2,18%) não concordaram em participar da pesquisa e somente 18 (9,78%) manifestaram o aceite, sendo: 16 (8,69%) fonoaudiólogos e dois (1,09%) médicos otorrinolaringologistas.

O tempo médio de preenchimento do questionário foi de 21 minutos. A caracterização do perfil dos participantes está apresentada na Tabela 2.

**Tabela 2:** Caracterização dos participantes da pesquisa (n=18)

**Informação**	**Descrição**	**n**	**%**
Idade	31 a 55	M = 39,1 anos	-
Sexo	Feminino	16	88,8
	Masculino	2	11,2
Região de atuação profissional	Norte	5	27,8
	Nordeste	4	22,2
	Sul	3	16,6
	Sudeste	5	27,8
	Centro-Oeste	1	5,6
Atuação com terapia fonoaudiológica para crianças e/ou adultos com deficiência auditiva	Não atuam	5	27,8
	Atuam com crianças	5	27,8
	Atuam com crianças e adultos	8	44,4
Locais de atuação	Hospitais	2	11,1
	Clínicas	10	55,5
	Universidades	3	16,7
	Outros	3	16,7
Conhecimento sobre o conteúdo avaliado	Sim	8	44,4
	Não	2	11,2
	Parcialmente	8	44,4

Legenda: n = número; % = porcentagem; M = média

Fonte: Elaborado pelos autores

A Tabela 3 demonstra os resultados referentes à avaliação do conteúdo do caderno de experiências.

**Tabela 3:** Respostas referentes à avaliação do conteúdo do material traduzido (n=18)

**Questões**	**Discordo totalmente**		**Discordo**		**Indiferente**		**Concordo**		**Concordo totalmente**	
	**n**	**%**	**n**	**%**	**n**	**%**	**n**	**%**	**n**	**%**
Considerando que o público alvo são fonoaudiólogos e familiares de crianças com DA, você considera que o texto apresentado no PDF é inteligível/compreensível?	0	0,0	0	0,0	1	5,5	10	55,6	7	38,9
O conteúdo está disposto de maneira lógica, facilitando a compreensão do mesmo?	0	0,0	1	5,5	0	0,0	12	66,7	5	27,8
A informação oferecida é suficiente para a elaboração de um caderno de experiências na rotina de crianças com deficiência auditiva?	0	0,0	0	0,0	2	11,1	9	50,0	7	38,9
O conjunto de imagens, figuras e vídeos são pertinentes ao conteúdo?	0	0,0	0	0,0	0	0,0	10	55,6	8	44,4

Legenda: n = número; % = porcentagem

Fonte: Elaborado pelos autores

Na Tabela 4 encontram-se as avaliações quanto à análise da qualidade visual do material traduzido - PDF, vídeos, imagens ilustrativas e legendas.

**Tabela 4:** Respostas referentes à avaliação da qualidade do material traduzido - PDF, vídeos, imagens ilustrativas e legendas (n=18)

**Questões**	**Péssima**		**Ruim**		**Regular**		**Boa**		**Excelente**	
	**n**	**%**	**n**	**%**	**n**	**%**	**n**	**%**	**n**	**%**
Como você classificaria a qualidade deste material (PDF e vídeos)?	0	0,0	0	0,0	0	0,0	7	38,9	11	61,1
Como você classificaria a qualidade das imagens ilustrativas do conteúdo disponibilizado em PDF?	0	0,0	0	0,0	1	5,5	9	50,0	8	44,5
Como você classificaria a qualidade dos vídeos e suas respectivas legendas?	0	0,0	0	0,0	2	11,1	11	61,2	5	27,7

Legenda: n = número; % = porcentagem

Fonte: Elaborado pelos autores

A importância do caderno de experiências na rotina clínica está descrita na Tabela 5.

**Tabela 5:** Respostas referentes à importância do material (n=18)

**Questão**	**Nem um pouco importante**		**Pouco importante**		**Razoavelmente importante**		**Muito importante**		**Extremamente importante**	
	**n**	**%**	**n**	**%**	**n**	**%**	**n**	**%**	**n**	**%**
Como você classifica o material elaborado?	0	0,0	0	0,0	1	5,5	7	38,9	10	55,6

Legenda: n = número; % = porcentagem

Fonte: Elaborado pelos autores

No presente estudo, o percentual de concordância no Índice de Validade de Conteúdo (IVC), foi de 94,48%.

## DISCUSSÃO

O caderno de experiências apresentado neste artigo teve como objetivo oferecer aos profissionais da área da fonoaudiologia mais uma possibilidade de recurso terapêutico direcionado ao desenvolvimento das habilidades auditivas e de linguagem de crianças com deficiência auditiva, de forma acessível, gratuita e *online*. Buscou-se, também, incentivar o conhecimento, o acesso, o interesse, a adesão e o envolvimento familiar no processo terapêutico, tanto em ambiente clínico quanto domiciliar, a partir dos vínculos construídos entre terapeuta, paciente e família, decorrentes das estratégias exploradas por meio do caderno de experiências.

A avaliação da validade de materiais da área da saúde, tem por objetivo facilitar a atuação dos profissionais no que diz respeito à promoção da saúde, a orientação e o apoio fornecidos à pacientes e familiares, com vistas à oferta de condições mais efetivas de cuidado, tratamento e reabilitação à população alvo^(19)^. A tradução do material foi bem avaliada pelos juízes, com taxas de concordância de validade maiores que 90%, conforme indicado pelo teste utilizado^(17)^. Tais taxas aplicam-se tanto aos aspectos de análise da linguagem, quanto à facilidade de entendimento, a atratividade do instrumento, a qualidade dos recursos audiovisuais e a ferramenta como um todo (Tabela 1).

A literatura especializada aponta alguns fatores relevantes à realização da validação de materiais na área da saúde, dentre eles, a possibilidade de obtenção de instrumentos mais completos, eficazes e com maior rigor científico, uma vez que a opinião de juízes contribui para o aperfeiçoamento da qualidade do material^(20)^. No presente estudo, as sugestões adicionais adquiridas por meio do questionário 1, consideradas relevantes, foram acatadas e ajustadas na ferramenta traduzida.

As informações disponíveis em materiais instrucionais em saúde só se tornam eficazes se compreendidas pelo público alvo^(21)^. Para isso, é necessário que o texto apresente um nível de legibilidade compatível com as capacidades cognitivas do leitor. Assim, ao produzir materiais com tais finalidades é de suma relevância que os profissionais da saúde direcionem atenção especial à utilização de recursos que facilitem a leitura e a compreensão do texto, maximizando o interesse do leitor tais como, a seleção e o uso de palavras, expressões técnicas e significados^(22)^. A qualidade dos textos disponíveis em ambiente *online* demandam cuidado ainda maior, tendo em vista a possibilidade e a velocidade de alcance a uma variedade de público com diferentes idades, escolaridade e níveis socioeconômicos^(13)^. Adicionalmente, nestes casos, é de responsabilidade dos fonoaudiólogos a publicação de informações seguras, confiáveis e fundamentadas em fontes e evidências científicas de qualidade^(12-14)^. A análise da legibilidade do texto traduzido no caderno de experiências classificou o material com nível fácil de leitura.

O uso de questionários *online* como instrumento de coleta de dados tem se tornado uma tendência atual, cada vez mais presente no meio científico, destacando-se como preferência tanto entre pesquisadores, quanto participantes^(23)^. Dentre as vantagens do uso deste material listam-se: o alcance global, a flexibilidade, a economia de tempo, as inovações tecnológicas, a facilidade de coleta e tabulação dos dados, o baixo custo, a obtenção de grandes amostras e o controle de preenchimento obrigatório. Em contrapartida ressaltam-se como desvantagens: a presença de *spam*, a seleção e a qualidade da amostra, a falta de habilidade dos respondentes, a necessidade de recursos tecnológicos, a impessoalidade e a baixa adesão^(24)^. Em relação ao último fator, a literatura aponta que questionários em formato *online* atingem, em média, 25% de adesão^(25)^.

A baixa adesão foi visualizada nesta pesquisa. Apesar da facilidade de acesso e da experiência dos convidados quanto ao uso da internet, a taxa de retorno dos profissionais mostrou uma limitação aos resultados, mesmo após o reenvio dos convites (três vezes no total), a adesão para a resposta ao questionário 2, foi de apenas 22 (11,96%) participantes. A justificativa à baixa adesão, pode estar pautada na falta de interesse e participação dos convidados, além do tempo necessário dispensado para a avaliação do conteúdo, o qual contou com a leitura do material em PDF (seis páginas), a análise dos vídeos (22,06 minutos), e a resposta ao questionário (média de 21 minutos). Apesar da baixa adesão dos profissionais convidados, o Índice de Validade de Conteúdo obtido revelou um percentual de 94,48%, indicando uma relação confiável entre o número de respondentes e as respostas obtidas.

Resultados semelhantes foram identificados em outras pesquisas relacionadas à internet. Um estudo^(26)^ investigou as competências de enfermeiras servidoras em instituições de ensino, quanto ao desempenho das funções essenciais de saúde pública, e apontou que, das 31 universidades convidadas, apenas seis (19,35%) retornaram o contato com indicação de profissionais *experts* em enfermagem. Destas, somente uma enfermeira (16,66%) respondeu ao formulário proposto. Outra pesquisa, propôs a aplicação de um questionário *online* com servidores técnicos-administrativos e chefes em educação. Nesta, as taxas de respostas obtidas foram de 20,78% (272), para os 1309 servidores convidados, e 13,85% (9), para os chefes respondentes do questionário^(27)^.

No que diz respeito ao conteúdo e coerência do material traduzido, vídeos e temática abordada, inteligibilidade e disposição do texto, recursos audiovisuais e informações para a composição do caderno de experiências, a maior parte dos avaliadores respondeu concordar ou concordar totalmente com os aspectos investigados (Tabela 3). Em análise adicional dissertativa, os participantes julgaram o material como prático, acessível e de fácil entendimento ao público, em decorrência da abordagem das diferentes fases e possibilidades para uma estimulação eficaz. De modo geral, os aspectos supracitados podem ser resumidos em três pontos principais: a acessibilidade, a facilidade de compreensão e uso do material. Sobre isso, a literatura aponta que conteúdos de orientação em saúde disponibilizados de forma *online* podem servir como recurso adicional de estimulação à criança, favorecer as redes de apoio terapêuticas e agregar ou disseminar novos conhecimentos. Tais aspectos são valiosos para a garantia da participação ativa da família no processo terapêutico^(28)^.

Na presente pesquisa a maioria dos participantes considerou a qualidade do material traduzido - PDF, vídeos, imagens ilustrativas e legendas, como boa ou excelente (Tabela 4). Ademais, de forma dissertativa, apontaram que a boa qualidade, ilustração e visibilidade das imagens facilitaram a compreensão e tornaram a leitura mais prazerosa. Os vídeos, por sua vez, foram descritos como bem produzidos, com boa qualidade e apresentação de situações práticas e reais, as quais enriqueceram todo o material. Sabendo que a literatura reconhece que o uso de ilustrações, vídeos e imagens pode favorecer a compreensão, a internalização e a aprendizagem de novos conteúdos^(29)^, considera-se que a forma como o material está organizado pode influenciar a adesão ao uso pelo público alvo.

Em relação à importância do material, 94,5% (n=17) considerou o caderno de experiências muito ou extremamente importante (Tabela 5). Além disso, todos os participantes fonoaudiólogos, referiram fazer uso do instrumento na prática clínica. Os terapeutas complementaram que este recurso terapêutico é muito importante e útil, pois, proporciona a estruturação da linguagem oral e escrita, estimula a criatividade e propicia um aumento nas relações familiares, com possibilidade de uso em todas as fases da terapia. Autores indicam que ferramentas adicionais voltadas à promoção do desenvolvimento comunicativo de crianças com deficiência auditiva, que possibilitam a adaptação às necessidades específicas de cada indivíduo, como o caderno de experiências, atuam como materiais ricos em estimulação, uma vez que ajustam-se aos diferentes contextos vivenciados pela criança^(30)^. Vale destacar que o potencial da utilização do caderno de experiências na terapia fonoaudiológica não se restringe à crianças com deficiência auditiva, podendo ser utilizado no processo terapêutico de crianças com outras alterações, por exemplo, distúrbios de linguagem^(2)^.

## CONCLUSÃO

A ferramenta “Como criar um caderno de experiências: histórias personalizadas em que a criança é a personagem principal”, foi avaliada como adequada quanto à tradução, com alta taxa de concordância de validade. A legibilidade revelou nível fácil de leitura. A maioria dos avaliadores classificou a qualidade e a identidade visual do conteúdo como excelente, de extrema importância e apropriado para disponibilização em *online*.

Tal material é de grande relevância, pois reúne informações práticas voltadas à criação de um caderno de experiências, bem como orientações quanto ao uso do material como recurso adicional para a estimulação do desenvolvimento das habilidades auditivas e de linguagem de crianças com deficiência auditiva.

Espera-se que com este estudo, fonoaudiólogos, profissionais da saúde, da educação, pais e/ou responsáveis e outros interessados tenham acesso a esta valiosa ferramenta terapêutica de forma *online* e gratuita, por meio do *website* Escutar Agora e Sempre da Cochlear®.
